# Role of Internal Jugular Vein Collapsibility Index in Predicting Post-spinal Hypotension in Pregnant Women Undergoing Cesarean Section: A Prospective Observational Study

**DOI:** 10.7759/cureus.39389

**Published:** 2023-05-23

**Authors:** Pharanitharan. N, Mamta Sinha, Mayank Kumar, Sarita Ramchandani, Monica Khetrapal, Khushbu Karoo, Bharath K Mesa

**Affiliations:** 1 Anaesthesiology, All India Institute of Medical Sciences, Raipur, Raipur, IND

**Keywords:** hypotension, spinal anaesthesia, internal jugular vein, ivc collapsibility index, cesarean section

## Abstract

Introduction

Post-spinal hypotension (PSH) frequently occurs in women undergoing cesarean section. In recent studies, Ultrasound-guided measurements of the internal jugular vein (IJV) have been reported to predict fluid responsiveness. We planned to evaluate the correlation between the internal jugular vein collapsibility index (IJVCI) and PSH in cesarean section patients.

Methods

Ninety-one parturients who underwent elective lower segment cesarean section with a singleton pregnancy were recruited. Preoperatively, patients were placed in a supine position with a 15-degree left lateral tilt. Maximum (at the end of expiration) and minimum (at the end of inspiration) IJV diameters (mm) and IJVCI were assessed using M-mode imaging during spontaneous and deep breathing. Spinal anaesthesia was performed at the L3-4 or L4-5 level. Systolic blood pressure, diastolic blood pressure, mean arterial pressure, heart rate, respiratory rate, and SpO2 were recorded from baseline till the delivery of the baby.

Results

Among 91 patients, 40 (45.5%) patients had at least one episode of hypotension. Demographic variables and baseline vitals were comparable between the hypotensive and normotensive groups (p>0.05). In spontaneous and deep breathing, IJV diameter at the end-expiration (IJVdmax), end-inspiration (IJVdmin), and IJVCI amongst both hypotensive and non-hypotensive pregnant women were statistically similar. Receiver Operating Characteristic (ROC) curve analysis showed that during spontaneous breathing, using a cut-off point of 29.5%, IJVCI had a sensitivity and specificity of 70% and 23%, respectively, for predicting PSH; whereas during deep breathing, IJVCI had a sensitivity and specificity of 77% and 27%, respectively, for predicting the same using a cut-off value of 37.5%.

Conclusion

We conclude that internal jugular vein parameters such as maximum diameter, minimum diameter, and IJVCI during spontaneous and deep breathing cannot be used as reliable predictors of post-spinal hypotension in pregnant patients undergoing elective cesarean section.

## Introduction

Parturients giving birth by cesarean section have increased in both developed and developing countries [1], due to its various maternal and foetal indications for reducing maternal and perinatal mortality. In parturients undergoing cesarean section, general and regional anaesthesia are the anaesthesia techniques of choice, with each having its indications [2] and complications. 

Spinal anaesthesia, is the preferred technique for elective cesarean sections as it avoids failed intubation risk, is easy to perform, and provides effective pain control [3]. However, post-spinal hypotension (PSH) is a common occurrence in obstetric anaesthesia practice accounting for up to 71% of cases [4].

Hypotension following spinal anaesthesia is mainly due to sympathetic blockade, which decreases venous return by reducing systemic vascular resistance. This is further exacerbated by aortocaval compression and increased sensitivity to local anaesthetics [5]. Hypotension, if uncorrected, can cause a decrease in uteroplacental perfusion, leading to adverse effects in the mother and fetal acidosis in the child [6]. Several approaches have been investigated to prevent and treat hypotension, including fluid preloading, co-loading with crystalloids or colloids, and using vasopressors such as phenylephrine and ephedrine [7].

Fluid overload is a risk with empirical volume loading and can be harmful in a pregnant woman with cardiac disease. Assessment of intravascular volume to predict PSH could improve clinical judgement-making and can guide towards early interventions to avoid severe complications for the mother and fetus.

Ultrasound measurement of the inferior vena cava (IVC) has been extensively studied for assessing the volume status of a patient. Studies have shown IVC diameter and inferior vena cava collapsibility index (IVCCI) as reliable predictors of intravascular volume status [8,9]. Still, IVC measurement is not feasible in all obese individuals and pregnant patients due to technical difficulties. Since the internal jugular vein (IJV) is superficial and easier to access compared to the IVC, several studies have been done measuring the IJV maximum and minimum diameters and collapsibility index in intensive care patients and have found it to be a helpful predictor in assessing the intravascular status [10-12]. Obstetric populations are different from general populations in terms of fluid management, and there are very few studies done on pregnant women measuring IJV parameters for predicting intravascular volume status [13-15].

The primary objective of this study was to evaluate the correlation of preoperative internal jugular vein collapsibility index (IJVCI) with episodes of intraoperative hypotension occurring after spinal anaesthesia until delivery of the baby.

## Materials and methods

This was a prospective, observational, single-cohort, clinical study. After obtaining clearance from the Institutional Ethics Committee of All India Institute of Medical Sciences, Raipur, (approval no. 1716/IEC-AIIMSRPR/2021), and registration with the Clinical Trials Registry of India (CTRI/2021/06/044798), parturients aged 18 to 40 years of age, with a singleton pregnancy of more than or equal to 37 weeks of gestation, of American Society of Anaesthesiologists (ASA) physical status II with no systemic illness and Lucas grade 3 and 4 of the urgency of cesarean section were included in the study.

The study excluded parturients with pregnancy-induced complications like gestational hypertension, patients with haemoglobin less than 7 gm/dl, hemoglobinopathies, peripheral vascular disease and associated cardiovascular or renal diseases.

Sample size calculation

The sample size was calculated considering the prevalence of PSH to be 71% (95% CI 58.0-81.8) as found in previous studies [4], with an allowable error of 10%. The total sample size came out to be 83. Considering a dropout of 10%, 91 patients were enrolled in the study.

Parturients undergoing elective cesarean section were kept fasting for six hours for solid foods and two hours for clear fluids before surgery. After performing a thorough pre-anaesthetic check-up, demographic data, and baseline hemodynamic parameters were noted.

IJVCI measurement

**Figure 1:**
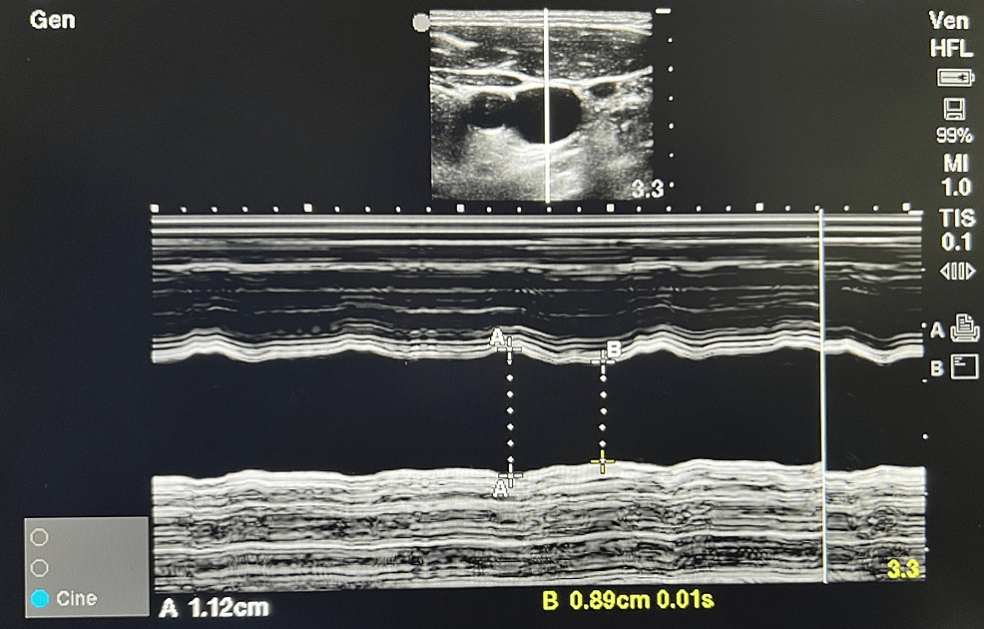
Ultrasound image of right IJV in M-mode IJV: Internal jugular vein A: End expiration and B: End inspiration

ASA standard monitors were attached in the operating room. Before administering spinal anaesthesia, patients were placed in the supine position with a 15-degree left lateral tilt. Patients were instructed to breathe normally at rest (spontaneous breathing), inhale deeply as much as possible, and exhale (deep breathing). The patients were then subjected to ultrasonography of the right side of the neck using a 6-13 MHz linear probe by a single anaesthesiologist well-trained and experienced in doing these measurements. With minimal contact, the probe was placed horizontally on the right side of the middle level of the thyroid cartilage, and a transverse view of the right IJV was obtained. In spontaneous and deep breathing, the maximum (at the end of expiration) and minimum (at the end of inspiration) IJV diameters (mm) and Collapsibility Index (%) = [(max IJV diameter - min IJV diameter)/max IJV diameter]*100 were assessed using M-mode imaging (Figure 1).

The anaesthesiologist performing the preoperative ultrasonography and the baseline parameter recording was not involved in the intra-operative monitoring and management. Co-loading with Ringer’s lactate at 10ml/kg was started, along with spinal anaesthesia placement. Under strict aseptic precaution, spinal anaesthesia was performed in the sitting position in L3-4 or L4-5 spaces by a 25-gauge Quincke’s needle, and 2.2ml of 0.5% bupivacaine heavy was given to the patient at a rate of 0.2 ml per second after verifying constant flow of cerebrospinal fluid. If the patient’s height was less than 150cm, then 2.0ml of 0.5% (heavy) bupivacaine was given. After 5 minutes of spinal anaesthesia induction, a sensory level of T6 was targeted and checked. Systolic blood pressure (SBP), diastolic blood pressure (DBP), mean arterial pressure (MAP), heart rate (HR), respiratory rate, and SpO2 were noted every 3 minutes for the first 20 minutes, and after that every 5 minutes, till the delivery of the baby. Hypotension was considered as a fall of MAP by 20 percent from baseline or < 65 mmHg. Episodes of hypotension were considered till the baby was delivered, to eliminate surgery-related causes of hypotension. Hypotension was treated with 6mg of mephentermine or 25 mcg of phenylephrine administered intravenously. Data collection was done till the delivery of the baby, and routine care was continued for the remainder of the surgery and in the post-operative period. The number of vasopressor doses administered to treat hypotension was recorded.

Statistical analysis

All the data collected were recorded in MS Excel and analysed in SPSS version 20 (IBM Corp., Armonk, NY). Continuous variables were checked for normality. Normally distributed data were presented as Mean ± Standard Deviation (SD), and the means between the two groups were compared by independent t-test. Non-normally distributed data were presented as Median (Interquartile Range) and represented through Box plots, compared with Mann Whitney U test. Variables repeatedly recorded with time were analysed by One Way Repeated measures ANOVA (General Linear Model). Categorical data were analysed by Chi-square test. The receiver operating characteristic (ROC) curve was plotted to calculate sensitivity and specificity. Logistic regression for binomial data was done to check the predicting p-value. A p-value of <0.05 was considered statistically significant.

## Results

Ninety-one patients were enrolled in the study who underwent elective lower segment cesarean section (LSCS) under spinal anesthesia. Among 91 patients, 40 (45.5%) patients had at least one episode of hypotension intraoperatively till the delivery of the baby. Demographic variables were comparable between hypotensive and normotensive groups (p-value > 0.05) (Table 1).

**Table 1:** Comparison of demographical variables between hypotensive and normotensive patients during the procedure SD (Standard Deviation), cm (centimeter), kg (kilogram), h (hours),  IQR (Inter Quartile Range)

	Parameters	All patients (n= 91)	Patients with hypotension		
S.NO			Yes	No	p-value
1.	Age (in years) (Mean ± SD)	28.73 ± 4.14	29.15 ± 4.22	28. 39 ± 4.08	0.391
2.	Height (cm) (Median {IQR})	157.00 (8.00)	157.00 (6.75)	155.00 (12.00)	0.434
3.	Weight (kg) (Median {IQR})	65.41 (10.00)	65.00 (11.5)	65.00 (12.00)	0.518
4.	BMI (kg/cm^2^) (Mean ± SD)	27.40 ± 3.64	27.46 ± 3.33	27.35 ± 3.90	0.885
5.	Abdominal Girth (cm) (Median {IQR})	99.00 (7.00)	98.50 (8.00)	99.00 (5.00)	0.904
6.	Period of fasting (h) (Median {IQR})	8.00 (2.00)	9.00 (2.00)	8.00 (2.00)	0.400

The mean age of patients was 28.73 ± 4.14 (24-32) years. Baseline vitals (SBP, MAP, HR) were statistically similar between hypotensive and normotensive groups (p-value >0.05) (Table 2).

**Table 2:** Comparison of baseline vitals between hypotensive and normotensive patients during the procedure SD: (Standard Deviation), SBP (Systolic Blood Pressure), DBP (Diastolic Blood Pressure), MAP (Mean Arterial Pressure), HR (Heart Rate), RR (Respiratory Rate), IQR (Inter Quartile Range)

	Parameters	Total n = 91	Patients with hypotension		p-value
s.no			YES	NO	
1.	Baseline SBP (mm hg) (Mean ± SD)	121.09 ± 9.90	119.50 ± 10.78	122.33 ± 9.01	0.177
2.	Baseline DBP (mm hg) (Mean ± SD)	75.26 ± 8.2	73.18 ± 9.00	76.90 ± 8.40	0.045
3.	Baseline MAP (mm hg) (Mean ± SD)	89.89 ± 8.79	88.25 ± 9.88	91.18 ± 7.68	0.115
4.	Baseline HR (beats per sec) (Mean ± SD)	90.88 ± 12.79	92.18 ± 13.59	89.86 ± 12.16	0.395
5.	Baseline RR (per min) [Median (IQR)]	20.00 (3.00)	20.00 (2.00)	20.00 (3.00)	0.909

Ultrasonographic observations were compared between the two groups. In spontaneous and deep breathing, IJV diameter at end-expiration (IJVdmax), IJV diameter at end-inspiration (IJVdmin), and IJVCI amongst both hypotensive and non-hypotensive pregnant women were statistically similar (Table 3,4). The Median (IQR) values of IJVdmax and IJVdmin measured during spontaneous and deep breathing did not show a statistically significant difference between the groups (Tables 3-4).

**Table 3:** Comparing the USG variables amongst the hypotensive and normotensive groups of patients in spontaneous breathing USG (Ultrasonography), Spont (Spontaneous), Max (Maximum), Min (Minimum), IJV (Internal Jugular Vein), IJVCI (Internal Jugular Vein Collapsibility Index)

Parameters	Total (n= 91)	Patient groups with hypotension		
		Yes	No	p-value
Spont. IJV Max diameter (Median {IQR})	0.770 (0.41)	0.790 (0.375)	0.710 (0.44)	0.535
Spont. IJV Min diameter (Median {IQR})	0.420 (0.35)	0.380 (0.43)	0.420 (0.32)	0.648
Spont. IJVCI (Mean ± SD)	41.65 ± 16.71	43.88 ± 18.62	39.82 ± 15.00	0.253

**Table 4:** Comparing the USG variables amongst the hypotensive and normotensive groups of patients in deep breathing USG (Ultrasonography), IJVCI (Internal jugular vein collapsibility index)

Parameters	Total (n =91)	Patient groups with hypotension			p-value
		Yes		No	
Deep Max diameter (Median {IQR})	0.640 (0.40)	0.700 (0.33)	0.570 (0.49)		0.264
Deep Min diameter (Median {IQR})	0.280 (0.27)	0.295 (0.25)	0.260 (0.28)		0.243
Deep IJVCI (Mean ± SD)	49.76 ± 17.22	50.50 ± 16.10	49.18 ± 18.18		0.718

The mean levels of the spontaneous and deep breathing collapsibility index (IJVCI) were higher in the hypotensive patients than in normotensive patients; however, the difference was not statistically significant.

Receiver operating characteristic (ROC) curve analysis was used to assess the IJVCI's diagnostic ability in predicting post-spinal hypotension. The area under the ROC curve for both IJVCIs (spontaneous and deep breathing) were 0.55 and 0.50 (p = 0.37 and p = 0.97 ), respectively, which was not statistically significant (Table 5).

**Table 5:** The characteristics of the ROC curve for IJVCI in spontaneous and deep breathing ROC (Receiver operating characteristic), AUC (Area under curve), Spont (Spontaneous), IJVCI (Internal Jugular Vein Collapsibility Index)

Variables	Cut off	Sensitivity (%)	Specificity (%)	AUC	p-value
Spont. IJVCI	29.5	70	23	0.554	0.377
Deep IJVCI	37.5	77	27	0.502	0.974

**Figure 2:**
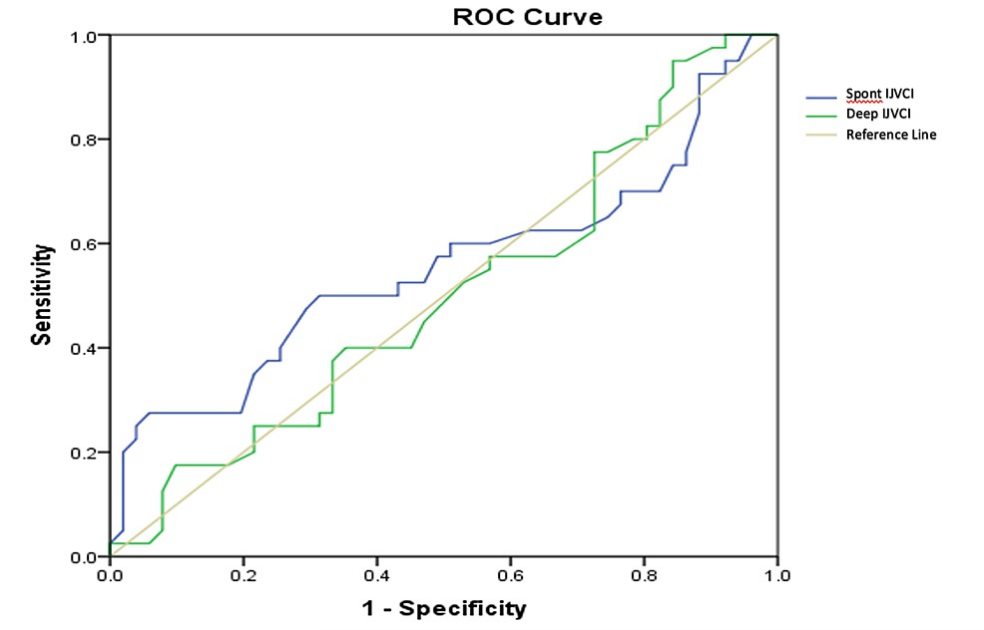
Receiver operating characteristics (ROC) curve of spontaneous and deep IJVCI for predicting post-spinal hypotension Spont (Spontaneous), IJVCI (Internal Jugular Vein Collapsibility Index)

During spontaneous breathing, using a cut-off point of 29.5%, IJVCI had a sensitivity and specificity of 70% and 23%, respectively, for predicting PSH, whereas during deep breathing, IJVCI had a sensitivity and specificity of 77% and 27%, respectively, for predicting the same using a cut-off value of 37.5% (Figure 2).

## Discussion

Hypotension after spinal anaesthesia is a well-known complication caused by sympatholysis, which is more aggravated in pregnant women than non-pregnant women due to their increased sensitivity to local anaesthetics and a 20% decrease in systemic vascular resistance due to the vasodilatory effects of progesterone and prostaglandins seen during pregnancy. In studies, hypotension after spinal anaesthesia during cesarean section has been shown to reduce uteroplacental blood flow leading to fetal acidosis [6].

Many strategies have been developed to assess intravascular volume status in pregnant patients before surgery, as blood pressure drops significantly in hypovolemic patients undergoing spinal anaesthesia. Measuring central venous pressure (CVP) through a central venous catheter was the gold standard for assessing intravascular volume status [16], but due to its invasiveness and the associated complications [17], it was quickly replaced by ultrasonography. New strategies for determining intravascular volume status with the use of ultrasonography have been developed, including measuring the diameters of the IJV, IVC, subclavian vein, and femoral vein [9].

Our study enrolled 91 healthy pregnant women (ASA II) scheduled for an elective cesarean section under spinal anaesthesia, and IJV measurements were taken before surgery. PSH was found in 44% of the patients, similar to the Elbardy et al. study [11], where the incidence of hypotension was around 47%. Our demographic variables did not differ significantly between normotensive and hypotensive patients. Frölich and Caton [18] found baseline HR to be a predictor of hypotension in patients undergoing caesarean delivery. However, in our study, baseline HR was not found to be a significant predictor of hypotension. Baseline diastolic blood pressure was significantly lower (p-value = 0.045) in the hypotensive group than in the normotensive group.

Panchal et al. [19] observed that the diameter of the IVC is a reliable predictor of hypotension in parturients undergoing caesarean delivery under spinal anaesthesia. However, there is difficultyin visualization of IVC in pregnant patients and Singh et al. [20] have reported a failure rate (non-visualization of IVC) of around 11% in their study in pregnant patients.

The gravid uterus during pregnancy, and the IVC's deep position, make ultrasonography visualisation of the IVC difficult for an anaesthesiologist. The superficial location and familiar anatomy of the IJV for the anaesthesiologist make it a good choice to study its measurements.

Jassim et al. [12] concluded that IJVCI can be used as a first-line approach for bedside non-invasive assessment of fluid status in critical patients. Elbadry et al. [11] conducted a similar study comparing the IJVCI and the IVCCI preoperatively to predict hypotension after spinal anaesthesia in pregnant patients undergoing caesarean delivery, and both IVCCI and IJVCI were found to be efficient and reliable tools in predicting post spinal hypotension. However, the difference in IJVCI parameters in our study was not statistically significant among the hypotensive and non-hypotensive groups.

The mean values of the IJVCI during spontaneous and deep breathing were higher in the hypotensive group in our study, which was similar to the findings in the study by Elbadry et al. [11]. The area under the curve for IJVCI during spontaneous and deep breathing was 0.55 (p-value = 0.377) and 0.52 (p-value = 0.974), indicating poor sensitivity and specificity for cut-off values of 29.5% and 37.5%, respectively. Our findings indicate that IJVCI is not a reliable predictor of PSH. However, Elbadry et al. found that a cut-off value of 38.5% for the IJV collapsibility index was an efficient predictor of post-spinal hypotension in pregnant patients.

Killu et al. [10] found that predicting hypovolemia in critically ill patients using cut-off value of 39% for IJVCI had a sensitivity of 87.5% and specificity of 100%. Okamura et al. [14] measured IJV in both supine and 10⁰ Trendelenburg positions before inducing general anaesthesia to predict hypotension, and they concluded that the area of IJV in the trendelenburg position was a reliable predictor of hypotension, in contrast, IJV parameters in the supine position were not significant.

Few recently published studies have found IVCCI as not a predictor of post-spinal hypotension in parturients undergoing elective cesarean delivery. Roy et al. [21] discovered an area under the curve (AUC) of 0.467 (p-value = 0.615) in a study on the IVCCI to detect PSH, which was not significant enough to name the IVCCI as a reliable predictor. Similarly, Singh et al. [20] measured ultrasonographic parameters of the IVC with and without wedge to predict hypotension after spinal anaesthesia in pregnant women undergoing caesarean section and found the area under curves of 0.46 and 0.38 respectively, concluding that IVCCI is not a reliable predictor of post-spinal hypotension in pregnant women.

Limitations

The findings are based on data from a single tertiary care institute and a homogeneous parturient study population. So, this data cannot be applied to general populations such as elderly patients, paediatric patients, or pregnant women with comorbidities. Our findings do not apply to parturients who are scheduled for emergency cesarean section as we recruited pregnant patients scheduled for elective cesarean section.

## Conclusions

Based on our findings, we conclude that IJV parameters such as maximum, minimum diameter, and IJVCI during spontaneous and deep breathing measured preoperatively by ultrasonography cannot be used as a reliable predictor of post-spinal hypotension in pregnant patients undergoing cesarean section. Though IJVCI is an easy, noninvasive method, it does not have the hypotension-predicting capability. Further studies with larger sample sizes are needed as this was a single-centre study.
